# Methods to Improve Joint Genetic Evaluation of Canine Hip Dysplasia Across BVA/KC and FCI Screening Schemes

**DOI:** 10.3389/fvets.2020.00386

**Published:** 2020-08-11

**Authors:** Shizhi Wang, Juliane Friedrich, Erling Strandberg, Per Arvelius, Pamela Wiener

**Affiliations:** ^1^Division of Genetics and Genomics, The Roslin Institute and Royal (Dick) School of Veterinary Studies, University of Edinburgh, Midlothian, United Kingdom; ^2^Department of Animal Breeding and Genetics, Swedish University of Agricultural Sciences, Uppsala, Sweden; ^3^Swedish Armed Forces Dog Training Centre, Märsta, Sweden

**Keywords:** best linear unbiased prediction, estimated breeding value, genetic correlation, genetic evaluation, hip dysplasia

## Abstract

The BVA/KC (British Veterinary Association/Kennel Club) and FCI (Fédération Cynologique Internationale) are the main screening schemes used to evaluate the status of canine hip dysplasia (HD) in Europe. Jointly utilizing HD records from both BVA/KC and FCI schemes could improve the reliability of genetic evaluation within and across countries. In this study, HD scores for German shepherd dogs (GSDs) in the UK (using the BVA/KC scheme) and Sweden (using the FCI scheme) were used to investigate how to better operate joint genetic evaluations across the two schemes. It was shown that under a bivariate model, which regarded BVA/KC and FCI scores as different traits, the estimated genetic correlations between the UK and Swedish GSD populations were the same when using BVA/KC total or worse hip scores and for single-country or joint analysis of both the UK and Swedish populations. Under a univariate model that converted BVA/KC scores into FCI scores, the predictability of estimated breeding values was slightly improved by performing a joint analysis.

## Introduction

Canine hip dysplasia (HD) is one of the most common orthopedic disorders in large and giant dog breeds (1). It was reported by the Orthopedic Foundation for Animals (OFA) (2) that 177 breeds were affected by HD, with the prevalence ranging from 0.9 to 75.3%, based on statistics of dogs born between 2011 and 2015. In veterinary practice, HD is diagnosed by radiographic screening and judged by the abnormal characteristics of hip joints. Currently, the BVA/KC (British Veterinary Association/Kennel Club) and FCI (Fédération Cynologique Internationale) are the main screening schemes used to evaluate HD status in Europe (3). Determined by the severity status of HD from normal to severe, aggregated scores of bilateral joints are given by 0–106 (0–53 for each joint) in the BVA/KC scheme and the grade of the worse joint is classified into A, B, C, D, or E in the FCI scheme. In addition to providing a veterinary diagnosis, HD scores/grades can be used to ensure pups are produced from healthy dogs and to calculate estimated breeding values (EBVs) of HD for genetic improvement.

Until now, national genetic evaluations based on HD screening schemes have been implemented in several European countries, (e.g. Sweden, Finland, and the UK). However, with the increasing number of exchanges of breeding animals and semen between European countries (4), joint genetic evaluation across countries should be considered as an approach to the genetic improvement of HD. The availability of EBVs calculated by joint genetic evaluation across countries would encourage and facilitate importation of dogs with high genetic merit. Another potential benefit of performing joint genetic evaluation of HD is increased genetic progress, particularly for countries with small dog populations (5). However, the reliability of joint genetic evaluation has been shown to be limited by genetic connectedness and genetic correlation between countries, especially for countries with different HD screening schemes (6).

In this study, using the German shepherd dog (GSD) as an example breed, BVA/KC scores in the UK and FCI grades in Sweden were utilized to investigate how to better operate joint genetic evaluations across countries with different screening schemes. First, genetic correlations between total or worse hip BVA/KC scores with FCI grades were estimated under a bivariate model (i.e., treating the UK and Swedish scores as two different traits). Secondly, instead of performing a bivariate model across HD schemes, BVA/KC scores for UK dogs were converted from continuous scores into categorical grades to perform a univariate model together with FCI grades for Swedish dogs (i.e., treating the data from the two countries as a single trait).

## Materials and Methods

### Data Preparation

**Figure 1:**
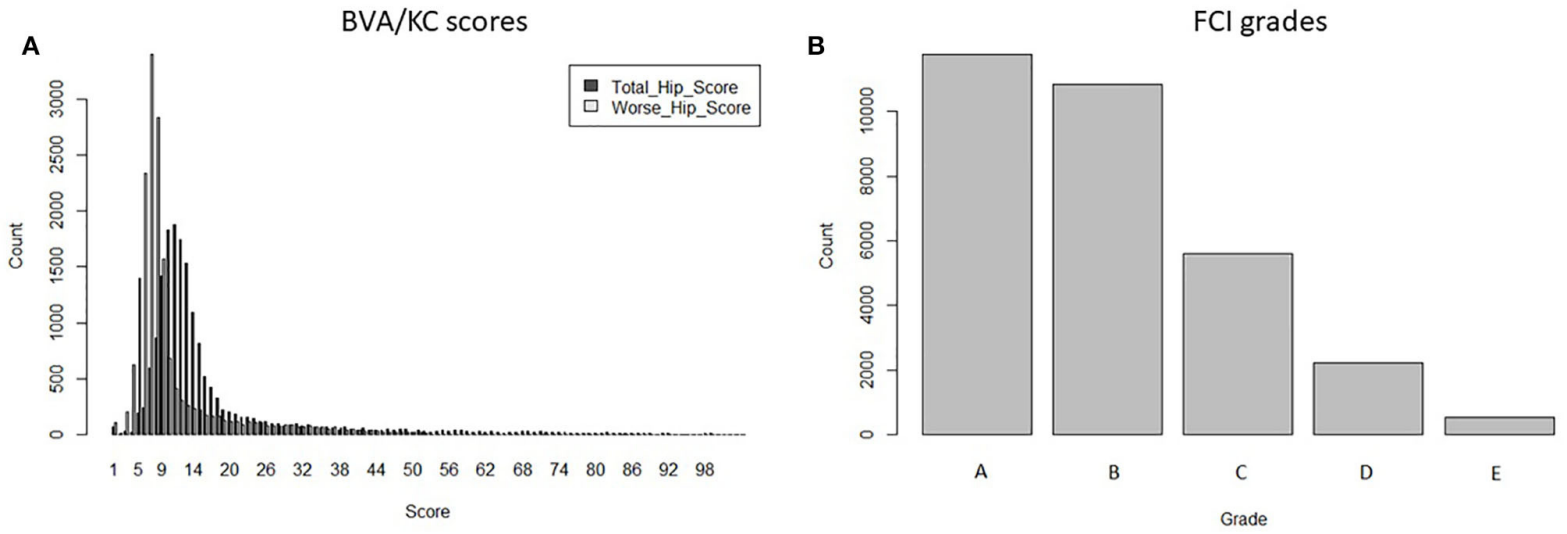
The distribution of hip dysplasia scores/grades recorded in the UK and Swedish German shepherd dog populations since 2000: **(A)** BVA/KC scores by total hip score and worse hip score in the UK; **(B)** FCI grades in Sweden.

Data used for analysis was provided by kennel clubs from the UK (The Kennel Club) and Sweden (Svenska Kennelklubben), including the pedigree and HD records of GSDs in each country. Dogs that occurred in both UK and Swedish pedigree databases (duplicated IDs) were detected by matching the individual's own and parental IDs. After replacing the duplicates with a unique ID, pedigrees of GSD from the two countries were merged into a combined database containing 877,280 registered animals. If HD was recorded with no screening date, recorded when the dog's age was <12 months or recorded before the year of 2000, then the HD record was removed from data analysis. The HD screening schemes in Sweden changed in 2000 (into FCI grades); thus, for simplicity, we chose to base our study on records since 2000. In total, 17,064 BVA/KC scores from the UK population and 30,909 FCI grades from the Swedish population were used to perform the study. There were no dogs with HD records in both screening schemes. The distribution of BVA/KC scores in the UK GSD population and FCI grades in the Swedish GSD population are shown in Figure 1.

For the bivariate model, both total hip scores (HS) and the hip scores for the worse hip (WS) from the BVA/KC scheme were transformed by natural logarithm into transformed total hip scores (THS) and transformed worse hip scores (TWS), as performed for the genetic evaluation of HD in the UK (7):

$$\begin{array}{l} THS=\;\ln\left(1+HS\right)\\ TWS=\;\ln\left(1+WS\right) \end{array}$$

**Figure 2:**
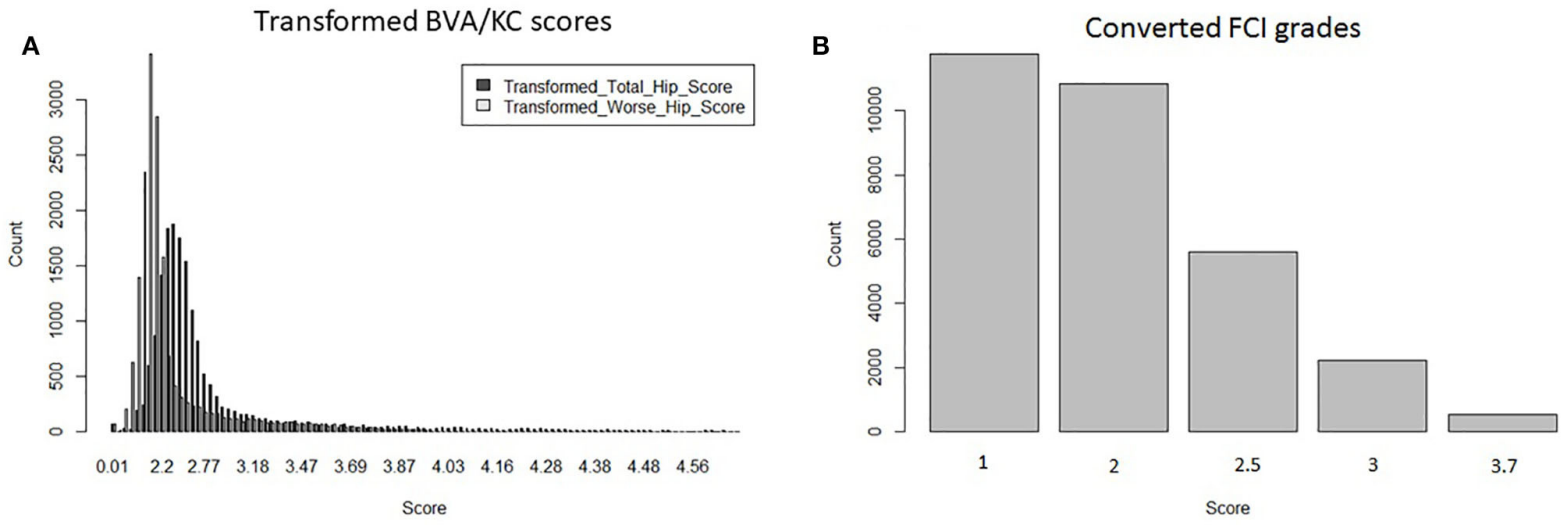
Distributions of transformed hip dysplasia scores/grades used in bivariate models: **(A)** transformed BVA/KC scores by total hip score and worse hip score in the UK population; **(B)** converted FCI grades in the Swedish population.

Hip grades A, B, C, D, and E of the FCI scheme were converted into scores 1.0, 2.0, 2.5, 3.0, and 3.7 (FCI_Five_), following the method used for genetic evaluation of HD in Sweden (8). The distribution of transformed BVA/KC scores (i.e., THS and TWS) and converted FCI grades (i.e., FCI_Five_) used in the bivariate model are shown in Figure 2.

**Figure 3:**
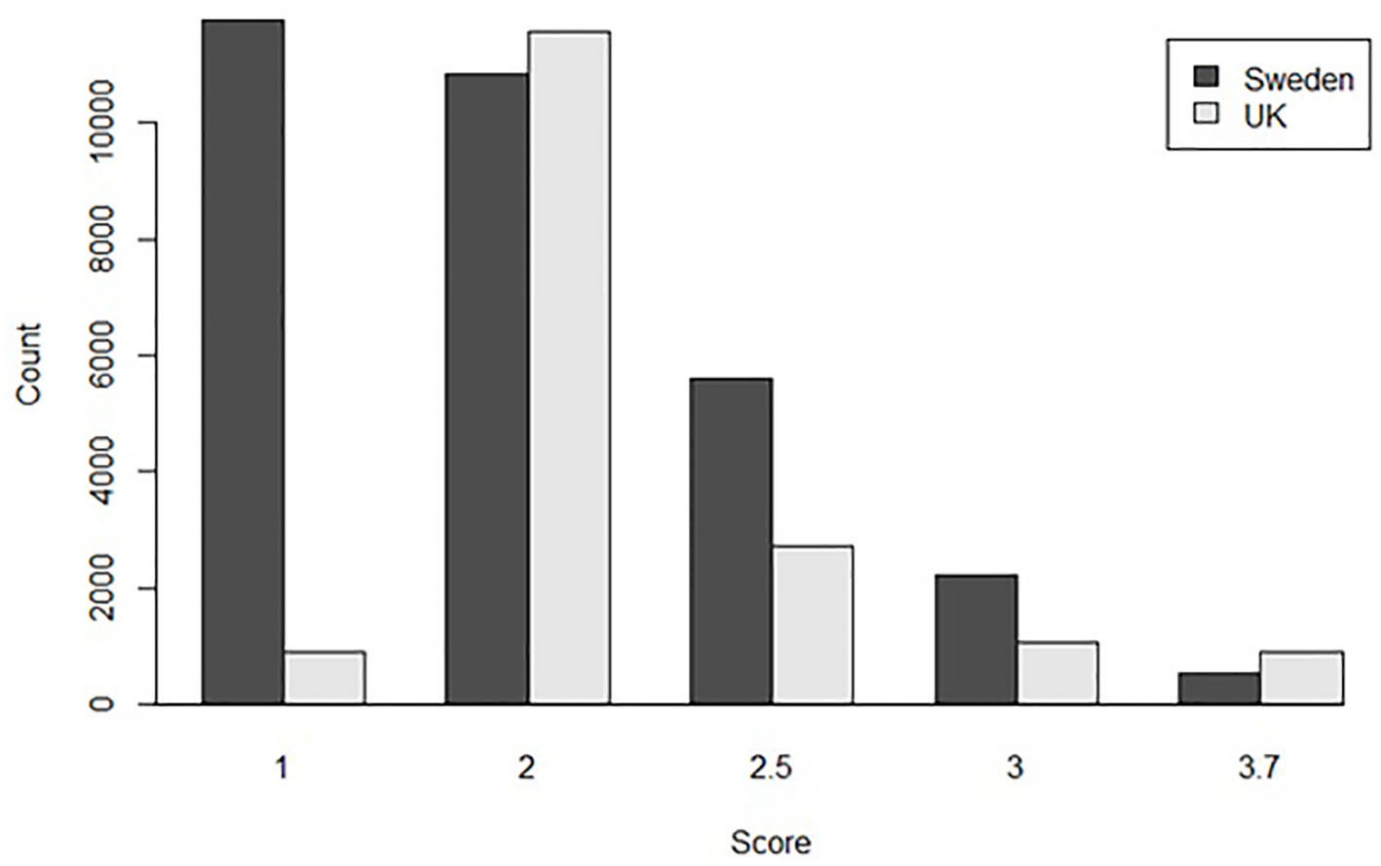
The distribution of converted hip dysplasia scores based on FCI five-level grades from the UK and Swedish populations used together in the combined univariate model.

For the univariate model, the method of conversion from BVA/KC scores into FCI grades involved transforming WS into the standard FCI five-grade scheme (A, B, C, D, and E) following a suggested conversion originally published by the BVA (0–3 = A, 4–8 = B, 9–18 = C, 19–30 = D, >30 = E) (9). In order to analyze UK and Swedish phenotypes together, BVA/KC scores of UK dogs were converted to FCI grades and then FCI grades of all dogs were converted to scores (as described above), which was defined as trait FCI_Five+Five_. The distributions of FCI_Five+Five_ from the UK and Swedish populations used in the univariate models are shown in Figure 3. In addition, a summary of the data used for the bivariate and univariate models is shown in Table 1.

**Table 1:** Hip dysplasia (HD) score/grades before and after data transformation/conversion in the UK and Sweden.

**Country**	**Screening scheme**	**Nr. of records**	HD score/grades*	After transformation/conversion*	
				**Bivariate model**	**Univariate model**
UK	BVA/KC	17,135	HS (0–106)	THS (0–4.67)	-
			WS (0–53)	TWS (0–2.67)	FCI_Five+Five_ (1.0/2.0/2.5/3.0/3.7)
Sweden	FCI	30,950	A/B/C/D/E	FCI_Five_ (1.0/2.0/2.5/3.0/3.7)	FCI_Five+Five_ (1.0/2.0/2.5/3.0/3.7)

* *HS, total hip score; WS, worse hip score; THS, transformed total hip score; TWS, transformed worse hip score; FCI_Five_, converted FCI five-level worse hip scores; FCI_Five+Five_, converted FCI five-level/five-level worse hip scores for UK/Swedish dogs*.

### Statistical Analysis

The program BLUPF90 (10) was used to run mixed linear models for both bivariate and univariate models using joint UK and Swedish population datasets; variance components were estimated by the average information-restricted maximum likelihood algorithm using pedigree information. The genetic models for bivariate models were formulated as below:

$$\begin{array}{lll} \left[\begin{array}{c} {\text{y}}_{BVA/KC}\\ {\text{y}}_{FCI} \end{array}\right] & = & \;\left[\begin{array}{cc} {\text{X}}_{BVA/KC} & 0\\ 0 & {\text{X}}_{FCI} \end{array}\right]\left[\begin{array}{c} {\text{b}}_{BVA/KC}\\ {\text{b}}_{FCI} \end{array}\right]\\ +\;\left[\begin{array}{cc} {\text{Z}}_{BVA/KC} & 0\\ 0 & {\text{Z}}_{FCI} \end{array}\right]\left[\begin{array}{c} {\text{a}}_{BVA/KC}\\ {\text{a}}_{FCI} \end{array}\right]\\ +\left[\begin{array}{cc} {\text{W}}_{BVA/KC} & 0\\ 0 & {\text{W}}_{FCI} \end{array}\right]\left[\begin{array}{c} {\text{u}}_{BVA/KC}\\ {\text{u}}_{FCI} \end{array}\right]\\ +\left[\begin{array}{c} {\text{e}}_{BVA/KC}\\ {\text{e}}_{FCI} \end{array}\right]\;\left(Bivariate\;model\right) \end{array}$$

where **y**_*BVA*/*KC*_ and **y**_*FCI*_ are the vectors of phenotypic values for transformed BVA/KC scores (THS or TWS) and converted FCI grades (FCI_Five_) in the bivariate models. **X**_*BVA*/*KC*_ (**X**_*FCI*_), **Z**_*BVA*/*KC*_ (**Z**_*FCI*_), and **W**_*BVA*/*KC*_ (**W**_*FCI*_) are incidence matrices, and **b**_*BVA*/*KC*_ (**b**_*FCI*_), **a**_*BVA*/*KC*_ (**a**_*FCI*_), and **u**_*BVA*/*KC*_ (**u**_*FCI*_) are solution vectors for fixed effects, additive genetic effects, and litter effects, respectively. The vectors of fixed effects for both countries consisted of sex, birth year, birth month, and age at screening. In the bivariate model, the vectors of additive genetic effects were assumed to follow a multivariate normal distribution with covariances as $\left[\begin{array}{c} {\text{a}}_{BVA/KC}\\ {\text{a}}_{FCI} \end{array}\right]\;\sim \;N\left(0,\;\left[\begin{array}{cc} \text{A}\sigma_{a_{BVA/KC}}^{2} & {\text{A}\sigma }_{a_{BVA/KC}a_{FCI}}\\ {\text{A}\sigma }_{a_{BVA/KC}a_{FCI}} & \text{A}\sigma_{a_{FCI}}^{2} \end{array}\right]\right)$, and the vectors of litter effects and residuals were assumed to follow multivariate normal distributions with no covariances as $\left[\begin{array}{c} {\text{u}}_{BVA/KC}\\ {\text{u}}_{FCI} \end{array}\right]\;\sim \;N\left(0,\;\left[\begin{array}{cc} \text{I}\sigma_{u_{BVA/KC}}^{2} & 0\\ 0 & \text{I}\sigma_{u_{FCI}}^{2} \end{array}\right]\right)$ and $\left[\begin{array}{c} {\text{e}}_{BVA/KC}\\ {\text{e}}_{FCI} \end{array}\right]\;\sim \;N\left(0,\;\left[\begin{array}{cc} \text{I}\sigma_{e_{BVA/KC}}^{2} & 0\\ 0 & \text{I}\sigma_{e_{FCI}}^{2} \end{array}\right]\right)$. The genetic models for univariate models were formulated as below:

$$\begin{array}{lll} {\text{y}}_{FCI} & = & {\text{X}}_{FCI}{\text{b}}_{FCI}+{\text{Z}}_{FCI}{\text{a}}_{FCI}+{\text{W}}_{FCI}{\text{u}}_{FCI}+{\text{e}}_{FCI}\\ \left(Univariate\;model\right) \end{array}$$

where **y**_*FCI*_ is the vector of combined HD phenotypes for UK and Swedish dogs (FCI_Five+Five_). **X**_*FCI*_, **Z**_*FCI*_, and **W**_*FCI*_ (**b**_*FCI*_, **a**_*FCI*_, and **u**_*FCI*_) are incidence matrices (solution vectors) for fixed effects, additive genetic effects, and litter effects in the univariate model, respectively. The vector of fixed effects in the univariate models consisted of sex, birth year, birth month, and age at screening, with an additional fixed effect, country, compared to the bivariate models. In the univariate model, the vector of additive genetic effects was distributed as ${\text{a}}_{FCI}\sim N\left(0,\;\text{A}\sigma_{a_{FCI}}^{2}\right)$, whereas litter effects and residuals were assumed to follow independent normal distributions ${\text{u}}_{FCI}\sim N\left(0,\;\text{I}\sigma_{u_{FCI}}^{2}\right)$ and ${\text{e}}_{FCI}\sim N\left(0,\;\text{I}\sigma_{e_{FCI}}^{2}\right)$, respectively.

Following the variance components estimation described above, heritabilities and genetic correlation (for the bivariate model) were calculated. For each trait/model, the Pearson correlation between EBVs and corresponding phenotypes was calculated to measure the predictability of EBVs, where THS was the corresponding phenotype for the EBV of THS (bivariate and univariate models), TWS was the corresponding phenotype for the EBV of TWS (bivariate model and univariate models), and FCI_Five_ was the corresponding phenotype for the EBVs of FCI_Five_ (bivariate and univariate models) and FCI_Five+Five_ (univariate model). Correlations between EBVs and corresponding phenotypes using single-population datasets were also calculated for THS and TWS in the UK population and for FCI_Five_ separately in the UK and Swedish populations to test whether the predictability was improved through joint-population analysis. Further details of the data used in the analysis are shown in Table 2.

**Table 2:** Descriptions of data analyzed in bivariate and univariate models.

**Model**	Trait*	**Number of records**	**Mean**	**S.D**.	**Range**
Bivariate analysis	THS	17,064	2.63	0.59	0.00–4.67
	TWS	17,064	2.16	0.57	0.00–3.99
	FCI_Five_	30,909	1.81	0.71	1.0/2.0/2.5/3.0/3.7
Univariate analysis	FCI_Five+Five_	47,973	1.94	0.68	1.0/2.0/2.5/3.0/3.7

* *THS, transformed BVA/KC total hip score; TWS, transformed BVA/KC worse hip score; FCI_Five_, converted FCI five-level worse hip scores; FCI_Five+Five_, converted FCI five-level/five-level worse hip scores for UK/Swedish dogs*.

## Results

### Estimation of Heritabilities and Genetic Correlation

In the bivariate models, estimated heritabilities for FCI_Five_ (Swedish dogs) were the same (0.27) whether the corresponding trait for UK dogs was THS or TWS (Table 3); the heritability of THS (0.41) was similar to that for TWS (0.39), which resulted from similar estimated genetic variances. The genetic correlation between FCI_Five_ and THS (0.67) was the same as that with TWS (0.67). In the univariate models, the estimated heritability for FCI_Five+Five_ was 0.23, which was lower than the estimate for FCI_Five_ in the bivariate model (0.27).

**Table 3:** Estimated variance components, heritability, and genetic correlation between BVA/KC and FCI scores (standard error) in the bivariate and univariate analysis.

**Model**	Trait*	**Animal**	**Litter**	**Residual**	**Total**	**Heritability**	**Genetic correlation**
Bivariate analysis	THS	0.14	0.02	0.18	0.34	0.41	0.67
		(0.01)	(0.00)	(0.01)	(0.02)	(0.02)	(0.21)
	FCI_Five_	0.14	0.05	0.32	0.50	0.27	
		(0.01)	(0.00)	(0.01)	(0.04)	(0.01)	
	TWS	0.12	0.02	0.17	0.32	0.39	0.67
		(0.01)	(0.00)	(0.01)	(0.02)	(0.02)	(0.22)
	FCI_Five_	0.14	0.05	0.32	0.50	0.27	
		(0.01)	(0.00)	(0.01)	(0.02)	(0.01)	
Univariate analysis	FCI_Five+Five_	0.10	0.03	0.29	0.42	0.23	-
		(0.00)	(0.00)	(0.00)	(0.01)	(0.01)	

* *THS, transformed BVA/KC total hip score; TWS, transformed BVA/KC worse hip score; FCI_Five_, converted FCI five-level worse hip scores; FCI_Five+Five_, converted FCI five-level/five-level worse hip scores for UK/Swedish dogs*.

### Correlation Between EBVs and Corresponding Phenotypes

In the UK population, the correlation between EBVs and corresponding phenotypes was slightly higher for THS (0.88) than that for TWS (0.87) in the bivariate models (Table 4). The correlations between EBVs and corresponding phenotypes of THS and TWS were the same for the single-population analysis (0.88 and 0.87) and joint-population analysis (0.88 and 0.87) using the bivariate model. Using the univariate model, the correlation between EBVs and corresponding phenotypes was slightly increased from 0.94 for single-population analysis (FCI_Five_) to 0.95 for joint-population analysis (FCI_Five+Five_).

**Table 4:** Correlation between EBVs and corresponding phenotypes in the UK population.

Single-population analysis**		Joint-population analysis**		
Trait*	**r(EBV, Pheno)**	**Model**	Trait*	**r(EBV, Pheno)**
THS	0.88	Bivariate	THS-FCI_Five_	0.88
TWS	0.87	Bivariate	TWS-FCI_Five_	0.87
FCI_Five_	0.94	Univariate	FCI_Five+Five_	0.95

* *THS, transformed BVA/KC total hip score; TWS, transformed BVA/KC worse hip score; FCI_Five_, converted FCI five-level worse hip scores; FCI_Five+Five_, converted FCI five-level/five-level worse hip scores for UK/Swedish dogs; r(EBV, Pheno), the Pearson correlation between EBVs and corresponding phenotypes*.

** *Single-population analysis only included UK data; joint-population analysis included both UK and Swedish data*.

For the Swedish population, the correlation between EBVs and corresponding phenotype, FCI_Five_, was 0.92 in the single-population analysis (Table 5). In the joint-population analysis, the correlations between EBVs and the corresponding phenotype, FCI_Five+Five_, were also 0.92 for both THS and TWS as correlated traits using the bivariate model. The correlation between EBVs and corresponding phenotype of FCI_Five+Five_ was slightly higher (0.94) for the joint-population analysis using the univariate model.

**Table 5:** Correlation between EBVs and corresponding phenotypes in the Swedish population.

Single-population analysis**		Joint-population analysis**		
Trait*	**r(EBV, Pheno)**	**Model**	Trait*	**r(EBV, Pheno)**
FCI_Five_	0.92	Bivariate	THS-FCI_Five_	0.92
		Bivariate	TWS-FCI_Five_	0.92
		Univariate	FCI_Five+Five_	0.94

* *THS, transformed BVA/KC total hip score; TWS, transformed BVA/KC worse hip score; FCI_Five_, converted FCI five-level worse hip scores; FCI_Five+Five_, converted FCI five-level/five-level worse hip scores for UK/Swedish dogs; r(EBV, Pheno), the Pearson correlation between EBVs and corresponding phenotypes*.

** *Single-population analysis only included Swedish data; joint-population analysis included both UK and Swedish data*.

## Discussion

By combining the UK and Swedish HD data (including pedigrees and phenotypic records), we investigated two main questions: whether THS or TWS is more appropriate for joint evaluation with FCI grades and whether BVA/KC scores for UK dogs converted to FCI grades are compatible with FCI grades for joint genetic evaluation across screening schemes, so that they can be treated as the same trait in a univariate model.

Heritabilities of HD estimated in this study ranged from 0.23 to 0.41, which were similar to the range previously estimated in the UK and Sweden (5, 7, 8, 11, 12). The estimated heritabilities for THS (0.41) and TWS (0.39) were higher than the reported heritability of THS (0.35) for GSDs in the UK (12). If all HD records (since the 1980's) were used for calculations, the heritability of THS was estimated as 0.35, the same as that previously estimated (12). This is because the total variance of HD recorded since 2000 was lower than the total variance of all HD records in the database, but the genetic variance was similar. In comparison, the heritabilities of FCI_Five_ and FCI_Five+Five_ estimated in this study were 0.27 and 0.23, respectively, which are close to 0.25 as previously estimated for the GSD population in Germany (13), likely due to the similar screening schemes in Sweden and Germany. Differences in the heritabilities for the traits FCI_Five+Five_ and FCI_Five_ reflect differences in the genetic variances for the joint and single populations.

The estimated genetic correlation (0.67) between transformed BVA/KC scores and converted FCI_Five_ grades for the GSD populations in this study was the same as that estimated for the Golden retriever breed (0.67) but lower than that for the Labrador retriever breed (0.82) when performing a joint genetic evaluation (bivariate model) between the UK and Sweden (5). This difference between breeds may be due to the fact that only HD data recorded from 2000 was used in this study and during this period from 2000 to present only 29 common sires (sires with offspring screened in both populations) existed between the UK and Swedish GSD populations. When the entire dataset of HD records for GSDs since 1980 was used, the number of common sires was 83 and the estimated genetic correlation was 0.80 between the UK and Swedish populations, suggesting that the number of records has a large influence on these estimates. Furthermore, the value of exchanging breeding animals will be greater for higher genetic correlation because the accuracy of EBVs across countries is “discounted” by the genetic correlation (accuracy of EBVs in original countries multiplied by the genetic correlation). Based on results from our previous study (6), very strong genetic correlations (>0.85) are necessary to ensure genetic progress equivalent to selection within an individual country when using foreign sires with EBV rankings in the top 50%, while only moderately high levels of genetic correlation (>0.70) are needed when using foreign sires with high EBV rankings, (e.g. in the top 10%). Based on the bivariate analysis of the UK (THS, TWS) and Swedish data (FCI_Five_), the correlations between EBVs and corresponding phenotypes for the UK population (THS, TWS) were the same for the single-population analysis (0.88, 0.87) and the joint-population analysis (0.88, 0.87). Similarly for the Swedish population, the correlations between EBVs and corresponding phenotypes of FCI_Five_ estimated from single- and joint-population analyses were both 0.92. The lack of improvement in performing a joint analysis may be due to two factors: (1) the number of phenotypes in each country was sufficient to guarantee high estimation reliability within each population, and (2) there were no dogs with screening records in both countries (i.e., no direct phenotypes were gained from performing a joint-population analysis). After converting BVA/KC scores into FCI grades (UK dogs) and performing genetic evaluation with FCI grades as a common trait (univariate analysis), the predictability in both the UK and Swedish populations was slightly improved by the addition of dogs from the other country, which suggests that there may be a benefit of “borrowing” BVA/KC scores from the UK to implement HD genetic evaluations. This may be particularly useful for countries (unlike Sweden) with few accumulated HD records.

For BVA/KC scores used in the UK population, our results suggest that neither THS nor TWS is a better trait on which to perform joint bivariate analysis with FCI grades. However, for the British national genetic evaluation under a univariate model using BVA/KC scores, the total hip score has previously been suggested to be a more appropriate trait for breeding against HD than the worse hip score due to the presumption that the differences between left and right hips derive from environmental influences rather than genetic effects (7). We only had access to data for the worse hip for the Swedish data in this study, but in the future, if Swedish data can be acquired for both hips, it would be valuable to further investigate this issue.

For both the UK and Sweden, under the joint-population analysis, the univariate model gives a higher correlation between EBVs and the corresponding phenotypes. However, for the UK population, this may in part be an artifact of the non-linear relationship between EBVs (based on natural log-transformed scores) and the original (non-transformed) BVA/KC scores (Figure S1), which does not apply to the Swedish data, where the data has not been transformed by logarithm. Furthermore, using the univariate model for both populations, the correlations between EBVs and corresponding phenotypes based on the joint-population analyses were slightly higher than those for single populations, demonstrating that a joint-population analysis would benefit genetic evaluation of HD in both populations.

Joint genetic evaluation across countries has been implemented in dairy cattle since 1983, using a well-defined Multiple Across-Country Evaluation (MACE) model (14), and much higher accuracy has been shown using joint genomic evaluation (15). In order to take an across-country approach for breeding against HD in dogs, the first technical challenge would be the unification of data from different screening schemes. In this study, we demonstrated that converting BVA/KC scores into FCI-like grades, which can then be used as additional phenotypes, could improve the predictability of breeding value estimation in countries using FCI grades (as for the Swedish population).

Recently, a small improvement in accuracy of genomic selection was seen for UK dogs in a joint genomic prediction of Norberg Angle score (one of diagnostic characteristics of HD) between US and UK Labrador retrievers (16); thus, future research could focus on joint genomic selection of HD between BVA/KC and FCI schemes. In addition, a genome-wide association study of canine behavior traits has been performed on a combined dataset of the UK and Swedish GSD populations (17), which suggests the potential to examine genetic factors influencing HD by performing a joint-population association analysis using a univariate model across countries/schemes (e.g., by converting BVA/KC scores in the UK population into FCI grades). This would give the potential to gain further insights into the genetic architecture of HD.

## Data Availability Statement

The data is not publically available because the authors don't hold permission to share it. It was provided by The Kennel Club and Svenska Kennelklubben. Requests to access the data should be addressed to these organizations directly.

## Ethics Statement

Radiographs were taken by veterinarians for submission to the British Veterinary Association/Kennel Club (UK) and Svenska Kennelklubben (Sweden) as part of the canine hip scoring schemes in the respective countries. We received approval for the study from the Veterinary Ethical Review Committee (VERC) and Human Ethical Review Committee (HERC) at the Royal (Dick) School of Veterinary Studies, The University of Edinburgh.

## Author Contributions

SW, ES, and PW designed the study. SW and JF analyzed the data. All authors contributed to the assembly of the data and preparation of manuscript.

## Conflict of Interest

The authors declare that the research was conducted in the absence of any commercial or financial relationships that could be construed as a potential conflict of interest.

## References

[1] Wilson B Nicholas FW Thomson PC Selection against canine hip dysplasia: success or failure? Vet J. (2011) 189:160–8. 10.1016/j.tvjl.2011.06.014 21727013

[2] OFA Orthopaedic Foundation for Animals. (2019). Available online at: https://www.ofa.org/diseases/breed-statistics#detail (accessed Jan 1, 2020).

[3] Flückiger M Scoring radiographs for canine hip dysplasia-the big three organisations in the world. Eur J Companion Anim Pract. (2007) 17:135–40.

[4] Wang S Leroy G Malm S Lewis T Strandberg E Fikse WF. Merging pedigree databases to describe and compare mating practices and gene flow between pedigree dogs in France, Sweden and the UK. J Anim Breeding Genet. (2017) 134:152–61. 10.1111/jbg.12242 27862377

[5] Wang S Leroy G Malm S Lewis T Viklund Å Strandberg E. Genetic correlations of hip dysplasia scores for Golden retrievers and Labrador retrievers in France, Sweden and the UK. Vet J. (2017) 226:51–6. 10.1016/j.tvjl.2017.07.006 28911842

[6] Wang S Strandberg E Viklund Å Windig JJ Malm S Lewis T. Genetic improvement of canine hip dysplasia through sire selection across countries. Vet J. (2019) 248:18–24. 10.1016/j.tvjl.2019.03.009 31113557

[7] Lewis TW Blott SC Woolliams JA Genetic evaluation of hip score in UK Labrador *Retrievers*. PLoS ONE. (2010) 5:e12797 10.1371/journal.pone.0012797 21042573 PMC2962628

[8] Malm S Fikse WF Danell B Strandberg E. Genetic variation and genetic trends in hip and elbow dysplasia in Swedish Rottweiler and Bernese mountain dog. J Anim Breeding Genet. (2008) 125:403–12. 10.1111/j.1439-0388.2008.00725.x 19134076

[9] BVA British Veterinary Association. (2019). Available online at: http://www.wheatenhealthinitiative.com/Pages/pdf%20files/hip-schemes.pdf (accessed May 13, 2020).

[10] Misztal I Tsuruta S Strabel T Auvray B Druet T Lee DH BLUPF90 and related programs (BGF90). Proc 7th World Congress Genetics Appl Livestock Prod. (2002) 33:743–4.

[11] Sánchez-Molano E Pong-Wong R Clements DN Blott SC Wiener P Woolliams JA. Genomic prediction of traits related to canine hip dysplasia. Front Genet. (2015) 6:97. 10.3389/fgene.2015.00097 25821457 PMC4358223

[12] Lewis TW Blott SC Woolliams JA. Comparative analyses of genetic trends and prospects for selection against hip and elbow dysplasia in 15 UK dog breeds. BMC Genet. (2013) 14:16. 10.1186/1471-2156-14-16 23452300 PMC3599011

[13] Stock KF Klein S Tellhelm B Distl O. Genetic analyses of elbow and hip dysplasia in the German shepherd dog. J Anim Breeding Genet. (2011) 128:219–29. 10.1111/j.1439-0388.2010.00901.x 21554416

[14] Schaeffer LR Robinson A Chesnais J Wilmink H Wiggans G Rozzi P Multi-trait, across country evaluation of dairy sires. Interbull Bulletin. (1993) 8:887–94.

[15] VanRaden PM Sullivan PG. International genomic evaluation methods for dairy cattle. Genet Selection Evol. (2010) 42:7. 10.1186/1297-9686-42-7 20193071 PMC2842236

[16] Edwards SM Woolliams JA Hickey JM Blott SC Clements DN Sánchez-Molano E. Joint genomic prediction of canine hip dysplasia in UK and US labrador retrievers. Front Genet. (2018) 9:101. 10.3389/fgene.2018.00101 29643866 PMC5883867

[17] Friedrich J Strandberg E Arvelius P Sánchez-Molano E Pong-Wong R Hickey JM. Genetic dissection of complex behaviour traits in German Shepherd dogs. Heredity. (2019) 123:746–58. 10.1038/s41437-019-0275-2 31611599 PMC6834583

