# Supplementary material for: Methods to Improve Joint Genetic Evaluation of Canine Hip Dysplasia Across BVA/KC and FCI Screening Schemes
Source: Front Vet Sci. 2020 Aug 11;7:386. doi: 10.3389/fvets.2020.00386 (PMC7432227; doi:10.3389/fvets.2020.00386)
Supplement: Supplementary file 1 [file Image_1.pdf]

Supplementary materials

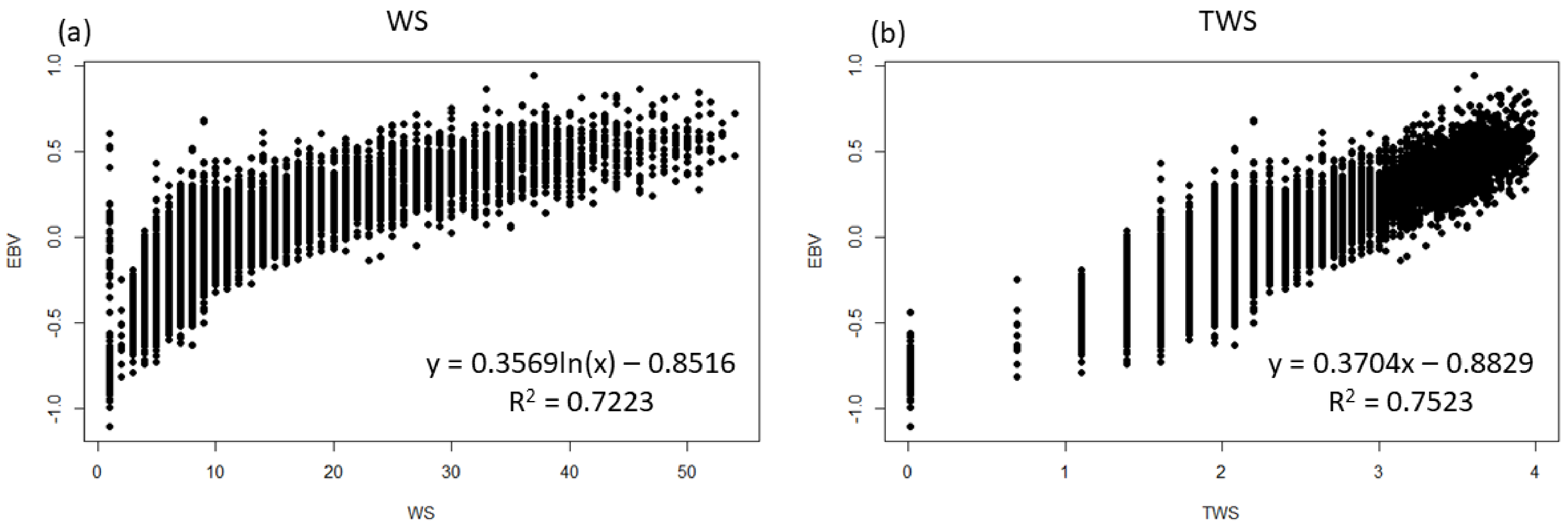

Figure S1. Scatterplots between EBVs estimated for the UK dogs using the bivariate model (joint-population analysis) with the corresponding phenotypes (a) un-transformed worse hip scores (WS) and (b) transformed worse hip scores (TWS).
